# Novel *Clostridium difficile* Anti-Toxin (TcdA and TcdB) Humanized Monoclonal Antibodies Demonstrate *In Vitro* Neutralization across a Broad Spectrum of Clinical Strains and *In Vivo* Potency in a Hamster Spore Challenge Model

**DOI:** 10.1371/journal.pone.0157970

**Published:** 2016-06-23

**Authors:** Hongyu Qiu, Robyn Cassan, Darrell Johnstone, Xiaobing Han, Antony George Joyee, Monica McQuoid, Andrea Masi, John Merluza, Bryce Hrehorak, Ross Reid, Kieron Kennedy, Bonnie Tighe, Carla Rak, Melanie Leonhardt, Brian Dupas, Laura Saward, Jody D. Berry, Cory L. Nykiforuk

**Affiliations:** Cangene Corporation, a subsidiary of Emergent BioSolutions Inc., 155 Innovation Drive, Winnipeg, MB, R3T 5Y3, Canada; University of Arizona, UNITED STATES

## Abstract

*Clostridium difficile* (*C*. *difficile*) infection (CDI) is the main cause of nosocomial antibiotic-associated colitis and increased incidence of community-associated diarrhea in industrialized countries. At present, the primary treatment of CDI is antibiotic administration, which is effective but often associated with recurrence, especially in the elderly. Pathogenic strains produce enterotoxin, toxin A (TcdA), and cytotoxin, toxin B (TcdB), which are necessary for *C*. *difficile* induced diarrhea and gut pathological changes. Administration of anti-toxin antibodies provides an alternative approach to treat CDI, and has shown promising results in preclinical and clinical studies. In the current study, several humanized anti-TcdA and anti-TcdB monoclonal antibodies were generated and their protective potency was characterized in a hamster infection model. The humanized anti-TcdA (CANmAbA4) and anti-TcdB (CANmAbB4 and CANmAbB1) antibodies showed broad spectrum *in vitro* neutralization of toxins from clinical strains and neutralization in a mouse toxin challenge model. Moreover, co-administration of humanized antibodies (CANmAbA4 and CANmAbB4 cocktail) provided a high level of protection in a dose dependent manner (85% versus 57% survival at day 22 for 50 mg/kg and 20 mg/kg doses, respectively) in a hamster gastrointestinal infection (GI) model. This study describes the protective effects conferred by novel neutralizing anti-toxin monoclonal antibodies against *C*. *difficile* toxins and their potential as therapeutic agents in treating CDI.

## Introduction

*Clostridium difficile* (*C*. *difficile)* is a Gram-positive, spore-forming anaerobic bacillus responsible for over 25% cases of antibiotic-associated diarrhea [1]. The prevalence of *C*. *difficile* associated infections (CDI) has increased significantly concomitant with the widespread usage of broad-spectrum antibiotics which suppress the normal microflora of the gut. In the US, CDI associated hospital stays increased 4 fold from 1993 to 2009, reaching 336,600 cases, or 0.9% of all hospital stays in 2009 [2,3]. Moreover, CDI related mortality rate was 9.1% of CDI inpatients. In Europe, the CDI related hospital admission was 0.23% [4] across multiple country hospital survey participants with a reported 8.8% related mortality rate. The enormous healthcare burden translates to an approximate annual cost of $8.2 billion [3] to treat hospitalized CDI in USA alone.

The severity of CDI ranges from asymptomatic carriage to diarrhea to life-threatening pseudomembranous colitis and fulminant colitis (toxic megacolon) [5,6]. Aside from age (>65 yr), a number of factors are recognized as predisposing individuals to the development of CDI including antineoplastic medications, prolonged hospitalization, gastrointestinal procedures, immune suppression, severe underlying illness and proton pump inhibitors [3,6,7], but most CDI manifests following antimicrobial treatment which disrupts the normally protective colonic microflora and allows for *C*. *difficile* colonization [7,8]. Since previous antibiotic administration is the primary risk factor of CDI, current treatment involves discontinuing inciting antibiotics and clearance of *C*. *difficile* bacteria with a limited choice of antibiotics including metronidazole, vancomycin or fidaxomicin [6,9]. Although vancomycin is effective for CDI cases, approximately 20–35% of infections relapse after antibiotic withdrawal [10,11]. This scenario is further complicated by the emergence and increased incidence of hypervirulent strains (BI/027/NAP1) [12–14]. The hypervirulent strains are responsible for severe infections associated with higher rates of recurrence and death [15]. Alternative treatments in development to reduce recurrent rates include many non-antibiotic biological therapies such as toxin specific monoclonal antibody cocktails [16] or non-specific polyclonal antibody administration (Immune Globulin Intravenous; IGIV) [17], active vaccination [18], non-toxigenic *C*. *difficile* prevention [19]), probiotics and fecal transplantation [20,21]. The increased prevalence of CDI with high recurrence rate following treatment indicate that current treatments are inadequate, and multifaceted approaches will be needed to treat CDI as a function of the complexity of patient’s pre-existing medical conditions, the diversity of disease manifestations, and the difficulties of outbreak prevention and transmission control.

Two large *C*. *difficile* specific exotoxins, toxin A (308KDa TcdA) and toxin B (270KDa TcdB), are the key virulence factors responsible for CDI establishment [22]. Both toxins share a high degree of amino acid sequence identity and similarity [23], giving rise to an arrangement of multidomain polypeptides which also share a considerable degree of structural homology and functional properties. In general, both have discrete functional domains including a C-terminal receptor-binding domain (fragment 4; F4), a central hydrophobic/transmembrane domain (fragment 2; F2), a proteolytic domain (fragment 3; F3) and an N-terminal glucosyltransferase enzymatic domain (GTD, fragment 1; F1) (S1 Fig) [24–28]. Both toxins modulate mammalian cell functions through inactivating small GTPases-Rho isoforms (Rho A, B and C), Rac, and Cdc42 following toxin-receptor binding, translocation into cytosol and proteolytic release of the functional glucosyltransferase intracellularly. As small GTPases are essential to maintaining the regular actin-based cytoskeleton of cells, TcdA and TcdB induce cell rounding and eventually cell death [22]. It has been found that both toxins can mimic the pathophysiological changes in *C*. *difficile* infected colitis by administration into animal intestine [29], including epithelial tight junction disruption and increased epithelial permeability, inflammation, cytokine and chemokine production [30]. Early studies with purified TcdA and TcdB, or isogenic *C*. *difficile* mutants that express functional TcdA or TcdB alone showed that both toxins were equally important in CDI pathogenesis [22,31], while recent studies with different animal models indicate that TcdB is more potent causing CDI [32–34].

Antibodies are the only therapeutic modality capable of completely neutralizing toxin. Neutralization of TcdA and TcdB with antibodies as a therapeutic approach has been found to protect animals against CDI [25,27,35–37]. Moreover, a limited clinical trial found that a cocktail of anti-toxin A and anti-toxin B monoclonal antibody treatment in combination with vancomycin significantly decreased the recurrence rate compared to vancomycin treatment alone [16]. Recently, two Phase III clinical evaluations showed promising results using monoclonal antibody treatments. For example that anti-toxin B monoclonal antibody treatment was an effective adjunctive therapy to prevent the recurrence of CDI [38,39]. In the current study, a panel of murine *C*. *difficile* toxin A- and toxin B-specific monoclonal antibodies (mAbs) were humanized and further evaluated for potency against *C*. *difficile in vitro* and *in vivo* in comparison to facsimiles of other mAb agents in development. From these studies, three humanized monoclonal antibodies demonstrated neutralizing capacity against toxins from a diverse selection of *C*. *difficile* clinical isolates, as well as demonstrated protection against B1 *C*. *difficile* infection in a primary spore challenge model in Golden Syrian Hamsters. Based on these findings humanized mAbs CANmAbA4, CANmAbB4 and CANmAbB1 retain bioactivity of the parental murine mAbs and merit further development as potential therapeutic agents in CDI treatment.

## Results

### Humanized mAbs against *Clostridum difficile* TcdA (CANmAbA4) and TcdB (CANmAbB4 and CANmAbB1)

Prior to the humanization process, selected murine mAbs demonstrated toxin specificity (did not cross react) and recognized unique epitopes as assessed by eptiope binning and/or competitive ELISA [40,41] in comparison to mAbs described in the literature (S1 Table). Following humanization, purified anti-toxin mAbs were tested to confirm identity and binding characteristics by ELISA (S2 Fig) and neutralization of TcdA/TcdB *in vitro* (S3 Fig). Combined with these evaluations, the affinity of humanized variants had been retained as determined by biolayer interferometry (Table 1). At this point, the humanized anti-TcdA monoclonal antibody was designated CANmAbA4, while humanized anti-TcdB mAbs were designated CANmAbB1 and CANmAbB4 and advanced for further evaluation.

**Table 1:** Summary of humanized (IgG1/κ) anti-toxin mAbs and murine progenitor from which they were derived.

Designate	CANmAbA4		CANmAbB4		CANmAbB1	
**Clone**	Humanized (Hu-CAN20G2)	Murine (CAN20G2)	Humanized (HuCAN46G24)	Murine (CAN46G24)	Humanized (Hu-CAN46G13a)	Murine (CAN46G13a)
***In vitro* neutralizing purified toxin**	Protective	Protective	Protective	Protective	Partially protective at high dose	Not protective*
***In vivo* neutralization**	Protective	Protective	Protective	Protective	Reduced protection	Protective
**Epitope**	TcdAF4	TcdAF4	TcdBF4	TcdBF4	TcdBF1	TcdBF1
**Affinity (KD)**	3.32E-10 M	4.19E-12 M	1.89E-09 M	1.89E-09 M	2.16E-09 M	8.57E-09 M
**V-sequencing**	Verified	Verified	Verified	Verified	Verified	Verified

* murine mAb CAN46G13a does not demonstrate *in vitro* neutralization

### *In vivo* neutralization of recombinant expressed *C*. *difficile* toxins

**Fig 1:**
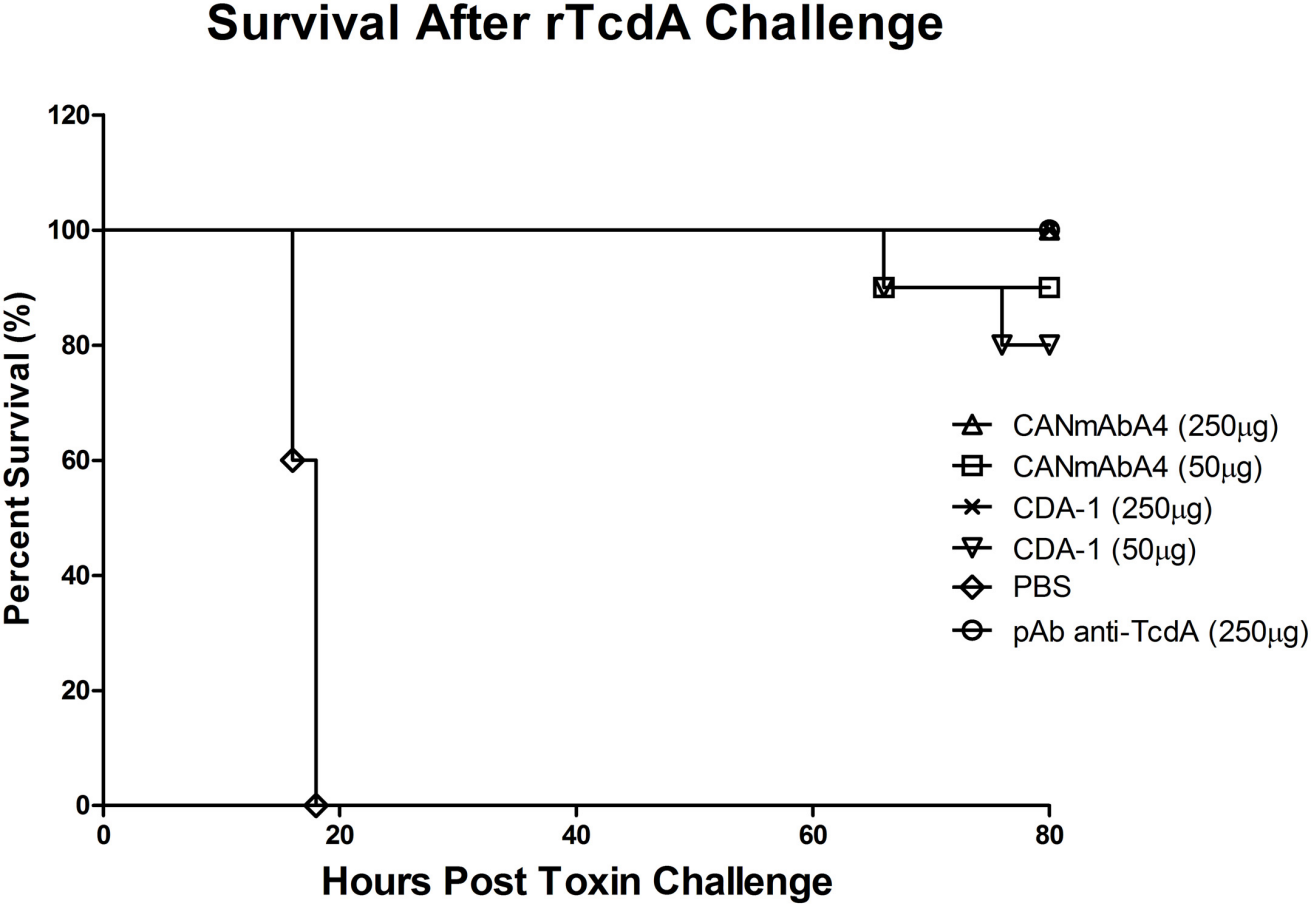
Survival after lethal rTcdA challenge in mice. Kaplan-Meier plot of survival following lethal challenge with rTcdA alone, or treatment with CANmAbA4 at either 250 μg or 50 μg dose in comparison to the CDA1 anti-TcdA and polyclonal (pAb anti-TcdA) control. Following lethal challenge, mice (n = 10) were monitored and sacrificed according to approved protocols. Statistical analysis (Log-rank Test) using GraphPad prism 5 indicated that all antibody treated groups had statistically significant higher survival rate compared to control group (rTcdA alone) (P<0.001). There is no significant difference in survival rate among antibody treated groups.

Humanized anti-Tcd mAbs were assessed in an *in vivo* murine toxin challenge model similar to that reported by Babcock et al. [25]. To date, only two monoclonal antibodies, CDA1 (anti-TcdA) and MDX1388 (anti-TcdB), have been tested as a combination or individually in clinical studies, with demonstrated efficacy [16]. Therefore these mAbs serve as suitable positive controls for our research (S1 Table) for *in vitro* and *in vivo* assessments where applicable (the in-house produced CDA1-comparator and MDX1388-comparator based on published sequence information (S1 Table) are referred to as CDA1 and MDX1388, respectively). Balb/c mice were treated with CANmAbA4, positive mAb control (CDA1), or with saline alone by intraperitoneal (i.p.) administration before challenge with a lethal dose of purified rTcdA (Fig 1). Animals were observed after toxin administration at designated times to monitor dosing for death/moribundity according to the approved protocol (AUP F10-040, Protocol Management and Review Committee (PMRC), University of Manitoba), (data not shown). Exposure to rTcdA alone in this model resulted in rapid death typically between 12–24 hours, and all animals in the saline group were moribund and euthanized within this time frame. All mice treated with CANmAbA4 and CDA1 control antibodies remained normal and active and survived to the end of the study at the higher 250 μg dose. At the lower dosing of 50 μg, 90% of CANmAbA4 treated mice and 80% within the CDA1 control group survived until the end of the study (Fig 1).

**Fig 2:**
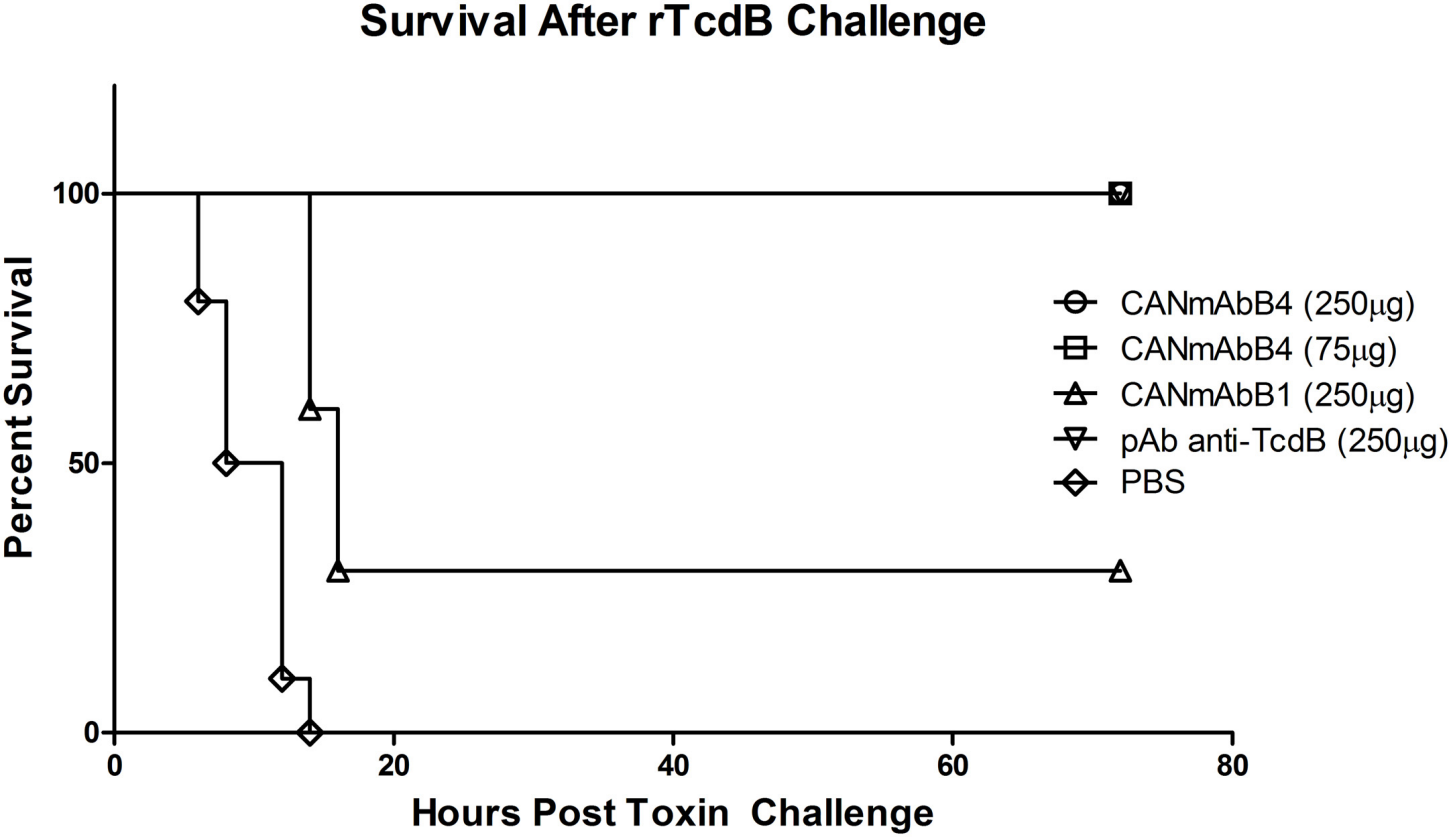
Survival after lethal rTcdB challenge in mice. Kaplan-Meier plot of survival following lethal challenge with rTcdB alone, or treatment with CANmAbB4 or CANmAbB1 at either 250 or 75 μg doses in comparison to the anti-TcdB rabbit polyclonal (pAb anti-TcdB) control. Following lethal challenge mice (n = 10) were monitored and sacrificed according to approved protocols. Statistical analysis (Log-rank Test) using GraphPad prism 5 indicated that all antibody treated groups had statistically significant higher survival rate compared to control group (rTcdA alone) (P<0.001). While CANmAbB4 treated animals (both 250 μg and 75 μg) showed statistically significant higher survival rate in comparison with CANmAbB1 (250 μg) (P<0.01).

Humanized anti-TcdB mAbs, were also tested for their *in vivo* protection against purified rTcdB challenge. Balb/c mice were treated with CANmAbB4, CANmAbB1, positive control (polyclonal antibody), or with saline alone by i.p. administration before challenge with a lethal dose of purified rTcdB (Fig 2). Exposure to rTcdB in this model resulted in rapid death between 12–24 hours. CANmAbB4 treated mice were fully protected at both doses, with 100% survival against lethal challenge with rTcdB over the 3 day study. In contrast, CANmAbB1, was only partially protective, with 30% of mice surviving at the 250 μg dose. The anti-TcdB mAb MDX1388 was not included as a control in this study as it does not provide neutralization of *in vivo* mouse toxicity using this assay [25].

### *In vitro* neutralization of culture supernatant toxins from clinical isolates

Neutralization across a number of toxins from clinical *C*. *difficile* strains from North America were assessed using humanized anti-Tcds. Based on preliminary tests, Vero and HT-29 cells lines demonstrated different sensitivity to toxin challenge (data not shown) and this was exploited to evaluate the efficacy of the humanized mAbs to inhibit *C*. *difficile* toxin dependent cytotoxicity. Supernatants from nine representative *C*. *difficile* clinical strains with variable PFGE/ribotypes and the reference strain, ATCC43255, were used with the xCELLigence^®^ system (ACEA Biosciences, San Diego, CA, USA), which provides a measurement of cell viability, morphology and integrity as an output of electrode impedance of cell culture surfaces. The toxin-neutralization ability of the mAbs was presented as EC_50_ (The mAb concentration which accounted for 50% neutralization of the toxicity from the culture supernatants). The lower EC_50_ indicates the better toxin-neutralizing efficacy. As shown in Table 2, CANmAbA4 demonstrated consistent neutralization of TcdA in all tested *C*. *difficile* isolates (note CF-2 lacks TcdA expression). In comparison to CDA1, a two way ANOVA analysis found that CANmAbA4 was significantly more effective in neutralizing TcdA across the tested isolates (ANOVA, p<0.05). For anti-toxin B mAbs, neutralization potency varied amongst the different strains tested. For non-hypervirulent (non-NAP-1/027) strains, CANmAbB4 and MDX1388 showed comparable neutralization of TcdB, whereby similar neutralization capacity (EC_50_) ranged from 100 to 500pM, but CANmAbB4 was less effective against the hypervirulent NAP1/027 strains in comparison with MDX1388. CANmAbB1, on the other hand, was less effective against the non-hypervirulent strains (in comparison to CANmAbB4 and MDX1388), but more potent amongst the hypervirulent NAP1/027 strains tested.

**Table 2:** *In vitro* neutralization of toxins from *C*. *difficile* clinical isolates by mAbs using xCELLigence^®^ test.

*C*. *difficile* Strain				Neutralization Titer (EC_50_, pM)				
				Antitoxin A mAbs		Antitoxin B mAbs		
PFGE type	strain	Ribotype	Toxin Phenotype	CDA1	CANmAbA4	MDX1388	CANmAbB4	CANmAbB1
non-NAP1	ATCC43255	Ribotype 087	0 (A+B+CDT-)	419.4	31.56^1^	113	114^4^	1820
	K14	Ribotype 053	0 (A+B+CDT-)	694.02	34.42^1^	228	180^4^	1845
	Y2*	-	0 (A+B+CDT-)	1208	28.85^1^	276	298^4^	1984
	B1	Ribotype 01, NAP2	0 (A+B+CDT-)	494.5	45.08^2^	417	165^4^	2120
	J9	Ribotype 01	0 (A+B+CDT-)	685	48.27^3^	144	137^4^	3425
	R23	Ribotype 012	0 (A+B+CDT-)	3419	23***	501	124^4^	2230
	CF2**	Ribotype 017	VIII (A-B+)	N/A	N/A	101	109^4^	15468
NAP-1	BI-1	Ribotype 027, NAP1	III (A+B+CDT+)	34933	58^2^	7717	NRV	5600^4^
	BI-6	Ribotype 027, NAP1	III (A+B+CDT+)	34067	49^2^	24100	NRV	2540^4^
	BI-17	Ribotype 027, NAP1	III (A+B+CDT+)	42400	54^1^	15875	NRV	4993^4^

Data are average of 3–4 experiments for each mAb/strain combination.

* Y2 is a common strain isolated from asymptomatic patients

** CF-2 is TcdA^-^TcdB^+^ strain, no toxin A production in the culture supernatant;

***, sample size too small.

NRV: non-reportable value.

^1,^ P<0.05;

^2,^ P<0.01;

^3,^ P<0.001;

^4,^ no significant difference.

N/A: not applicable.

### Passive protection from *C*. *difficile* spore challenge in Golden Syrian Hamster model

Previously, Babcock et al. [25] described an established hamster primary B1 infection model to determine the effectiveness of mAb combinations in preventing *C*. *difficile* infection in Golden Syrian Hamsters. In order to assess humanized anti-Tcds, a similar model was developed for B1 infections, with minor modifications. Briefly, daily i.p. injections of humanized mAbs were conducted 3 days prior to and on the day of intragastrically (i.g.) administrated *C*. *difficile* B1 spores, and the health and survival of hamsters monitored for 22 days. Twenty-four hours prior to spore challenge, hamsters were treated with clindamycin to disrupt and clear gut bacteria flora to enhance *C*. *difficile* spore infection. Based on *in vitro* neutralization results (Table 2), the EC_50_ of CANmAbB1 is approximately 12.8 times higher than that of CANmAbB4 in neutralizing B1 culture supernatant, indicating that CANmAbB4 is superior over CANmAbB1 against B1 derived TcdB, therefore the selection of humanized mAbs was limited to CANmAbA4/CANmAbB4 combinations at high and lower doses for demonstrating effects against B1 infection.

**Fig 3:**
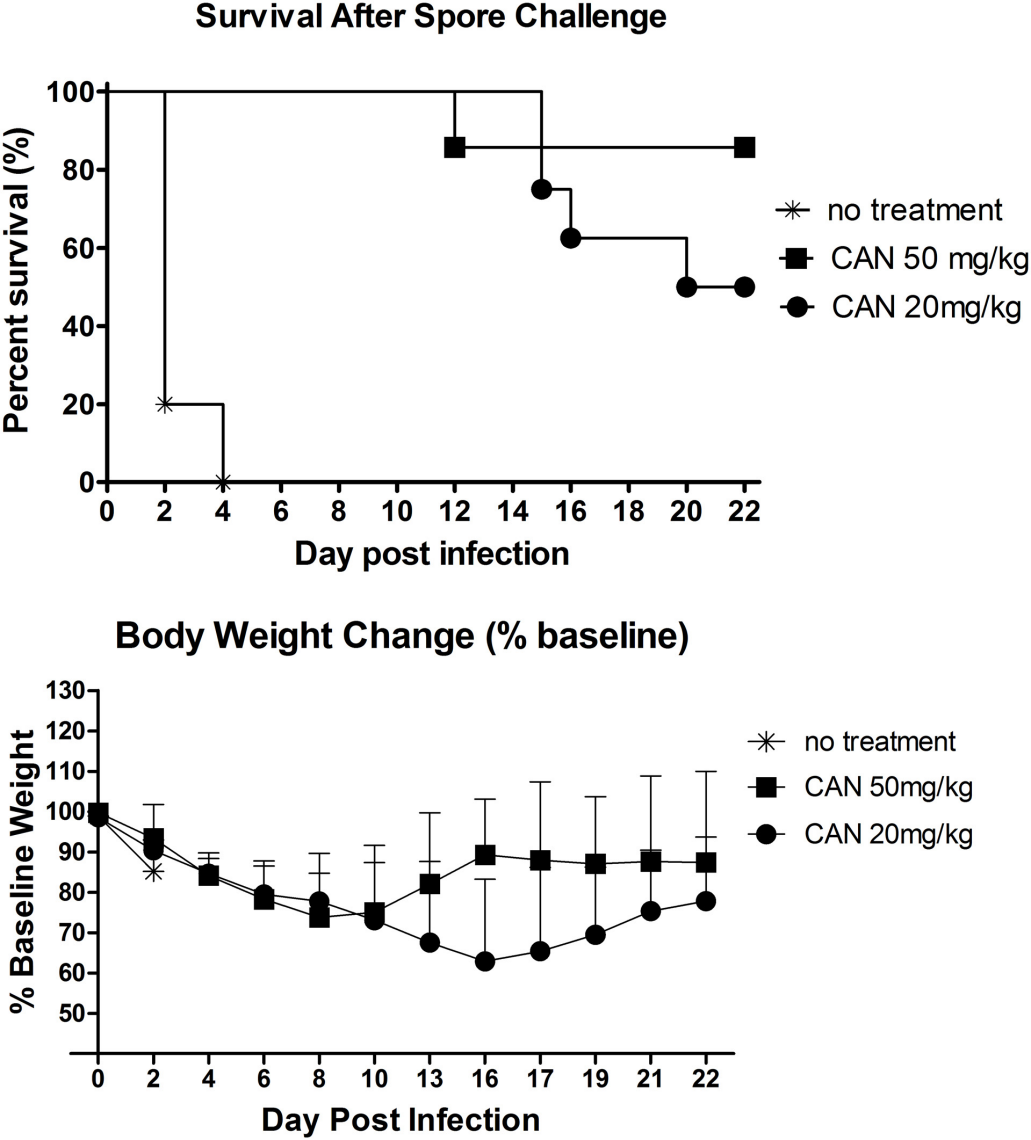
Protection of hamsters with humanized anti-toxin mAbs in primary oral gastrical infection model. Hamsters were treated with designated high or low doses of anti-toxin mAb combinations followed by B1 spore challenge. Animals were monitored for (A) survival and (B) body weight change for 22 days. Statistical analysis (Log-rank Test) using GraphPad prism 5 showed that both CANmAbs treated groups (50 mg/kg and 20 mg/kg) had significant higher survival rate in comparison with no treatment (PBS control) group (P<0.001). While the bodyweight changes between 50 mg/kg and 20 mg/kg groups were also significantly different (P<0.05) by one way ANOVA followed with Bonferroni’s multiple comparison test. For no treatment (n = 5), for 50 mg/kg treatments (n = 7) for 20 mg/kg treatments (n = 8). CAN: CANmAbA4/CANmAbB4 combination.

As shown in Fig 3a, the majority of animals within the untreated group (phosphate buffered saline (PBS) treatment) died within 48h after infection and had no survival 4 days post infection (DPI), which indicates spore infection was established successfully. For the CANmAb cocktail (CANmAbA4 and CANmAbB4) (50 mg/kg) treatments, only one hamster died of infection on 12 DPI and the remaining six hamsters (85%) all survived until the end of the study, of which four hamsters didn’t show any clinical signs in the last week of the study period. In comparison, the survival rates of the 20 mg/kg treatment group was lower than the 50 mg/kg dosage treatment group; two hamsters were euthanized 15 DPI, with another two euthanized/died 16 and 20 DPI, respectively, resulting in an overall survival of 50% in this group. The treated animals final survival rates were between 50% and 85%, dependent upon dosing, both of which are significantly different from the untreated control group (PBS treatment, P<0.001), and indicative of the protective function of toxin-specific antibody treatment. During the same period, hamster body weights (BW) were monitored and decreased significantly after infection in all control (for first 48 hours) and treatment groups (Fig 3b). For the CANmAb treatment at the higher dose (50 mg/kg), BW dropped to 73% of baseline on 8 DPI, then recovered to 87% of the baseline by the end of the study. For 20 mg/kg mAb treatments, BW dropped to about 65% of baseline on 16 DPI, they then started to recover and had regained 77% of original weight at the end of the study.

At the end of the experiment (22 DPI), all surviving animals were euthanized for necropsy and the gross pathology of the abdomens was performed (Data not shown). From this analysis, there were no significant differences in colon hemorrhage, edema and enlargement, or in the physical characteristics of the stool in the ceca between the different treatment groups. Additionally, circulating serum antibody levels were also measured in surviving animals through Magpix^®^ multiplex^™^ assay (see Materials and Methods) and were correlated with host resistance to spore challenge (S2 Table). The 22 DPI hamster serum samples still showed measurable anti-TcdA IgG antibody concentrations between 57–1992 nM and anti-TcdB IgG antibody concentrations ranged between 61–231 nM, indicating that the protection of CANmAbs in hamster may last several weeks.

## Discussion

To date, the most effective treatment of CDI patients is antibiotic treatment at the initial episode, but is often associated with a high rate of recurrence once antibiotic administration is discontinued [3,42]. Within this context, the increasing incidence of nosocomial and community acquired *Clostridium difficile* (*C*. *difficile)* infection (CDI) has accelerated the search and development of new therapies [43–45] to augment or combine with antibiotics. Since the *C*. *difficile* toxins, TcdA and TcdB, play a pivotal role in the progress of CDI [22] and antibiotics have no effect on the toxins, toxin neutralizing antibodies have been studied in animal models and shown protection against *C*. *difficile* infections [25,27,35]. Using combinations of anti-toxin A (CDA1) and toxin B (MDX1388) antibodies as adjunct treatment concomitant with vancomycin, significantly reduced the CDI relapse rate in a phase II clinical trial [16]. Two recently finished clinical studies (NCT01513239 and NCT01241552) also demonstrated that either anti-TcdA and anti-TcdB combination, or anti-TcdB alone, can significantly decrease the recurrence rate when administrated to patients during antibiotic therapy for *C*. *difficile* infection [38,39], indicating the potential advantage of anti-toxin antibody/antibiotic co-administration treatment over standard antibiotic therapy alone. In this study, we described the development of a series of IgG1/κ humanized antibodies with high affinity to *C*. *difficile* toxins. These antibodies showed broad neutralization of toxins from nine clinical isolated *C*. *difficile in vitro*. Moreover, CANmAbA4 and CANmAbB4, significantly prolonged and increased survival in a B1 *C*. *difficile* hamster primary infection model in comparison to the vehicle control group.

Antibodies were raised in mice to recombinant *C*. *difficile* toxin A (rTcdA) and toxin B (rTcdB) [40,41]. In the process of immunization and clone selection, we also compared the humoral responses generated by TcdA and TcdB, which are homologous toxins, sharing 49% identity and 63% similarity at the amino acid level. They belong to a family of large clostridial toxins, and display a multi-modular structure of the ABCD model (A activity; B binding; C cutting; D delivery). However, despite their similar structure, antibodies generated by one toxin had very low cross-reactivity with the other one. This is in agreement with others’ implicating a cross-neutralizing antibody will not likely be developed against both TcdA and TcdB [46,47]. TcdA and TcdB are large proteins (308 and 270 kDa respectively) with multiple epitopes and are excellent antigens for generating antibodies, but we observed that in the case of TcdB, most toxin-binding mAbs resulting from immunization were non-protective. Indeed, out of more than 2000 hybridoma clones screened, about 130 clones were identified to have TcdB-binding antibodies, but only 5 clones had *in vitro* and *in vivo* neutralizing abilities. The low success rate for isolating neutralizing anti-TcdB clones is not an uncommon phenomenon, and while hard to generalize it is still much lower than the reported 14% of protective mAbs generated from toxin immunizations [48]. Interestingly, similar difficulties of finding TcdB neutralizing activity was documented when llama immune phage display libraries were used to select TcdA and TcdB targeted single domain antibodies (VHH fragments), demonstrating a poor correlation between immunogenicity and functionality (induce neutralizing antibody) when TcdB was used as an antigen [49]. Alternatively, polyclonal antibody responses may be protective *in vivo*, but the low frequency of protective mAbs after TcdB immunization indicated the low level of neutralizing antibodies. Together these findings are an important consideration regarding vaccine studies. The toxins, especially the C-terminal CROP (combined repetitive oligopeptide) region of the toxins have been the traditional immunogenic choice for vaccine development. It’s important to take into consideration that TcdA and TcdB may induce different intensity and quality of immune responses. Indeed, in Phase I bivalent toxoid (A and B) vaccine trials, it was found that overall the immune response to toxin B was less than that observed to toxin A [50]. The independent antibody levels and neutralization abilities to TcdB might also partially explain the discrepancy of previous studies regarding which antibody is more associated with protection in patients. Our experience suggests that the anti-toxin humoral responses generated by immunization or infection need to be evaluated for both antibody titers and functionality [51–53].

We generated several different versions of humanized antibodies based on the lead candidates and tested *in vitro* neutralization capacity (S3 Fig). Both CANmAbA4 and CANmAbB4 strongly neutralized the toxin cytopathology and were selected for further analysis. Although CANmAbB1 showed weak neutralizing ability, it was immunologically distinct and recognized the epitope on TcdB fragment 1 instead of 4 (Table 1). It was noticed that a high amount of CANmAbA4 (about 500 ng/ml) was needed to neutralize the toxicity of TcdA in comparison with some published data (about 1–10 ng/ml) [35]. However, these two experiments used different cell lines, different sources of toxins and different methods to evaluate the neutralization, which makes direct comparison difficult. CANmAbA4 specifically recognizes a unique epitope on the receptor binding subdomain of *C*. *difficile* TcdA with subnanomolar affinity. CANmAbB4 specifically recognizes an epitope on the receptor binding subdomain of *C*. *difficile* TcdB, while CANmAbB1 specifically targets an eptiope on the glucosyltransferase subdomain, both with nanomolar affinity. In order to assess neutralization of systemic cytotoxicity, mice were treated and challenged with rTcdA (Fig 1) or rTcdB (Fig 2). CANmAbA4 was fully protective at 250 μg dose, and survival rates were only slightly reduced (90%) at the 50 μg dose. In a similar manner, the positive control, CDA1 was also fully protective with observed reductions to 80% at the 50 μg dose. Previously reported neutralization with CDA1 was approximately 70% when similar concentrations of mAb treatment and toxin were used [25], however it should be noted that this reported value was derived across multiple experiments under treatments of 100 to 250 μg CDA1 with 100 ng of toxin, and therefore differences in toxicity of the lot of TcdA used, purity of the CDA1 preparations, formulation of the antibody, glycoprofile, and fully assembled mAb may contribute to this apparent discrepancy. For humanized anti-toxin B, polyclonal antibodies raised against TcdB were used as the positive control, as according to the authors, MDX1388 did not effectively neutralize *in vivo* toxicity in a mouse toxin B challenge model [25]. In this respect, CANmAbB4 was fully protective at both the 250 and 75 μg dose, whereas CANmAbB1 was only partially protective (30%) at the higher dose. There has been an ongoing discussion in the field focused on the relative contribution of specific domains of toxin A and toxin B in mediating a protective response. Both the receptor binding domain and glucosyltransferase subdomains have proven most effective in raising neutralizing antibodies to TcdA and TcdB [25,27,35,54]. This reflects the functional, conformational and genotypic/phenotypic conservation of domains across different *C*. *difficile* Tcds. Although not completely delineated, it is speculated that *C*. *difficile* monoclonal antibodies raised against the receptor binding subdomains may neutralize the toxin by preventing binding to the cognate receptor of target cells, and therefore confer protection [25,55,56]. For antibodies raised against the glucosyltransferase subdomain, neutralization may occur partially by disrupting receptor binding through steric hindrance or block glucosyltransferase activity intracellularly. Another consideration facilitating the effectiveness of anti-Tcds are the constant regions involved in effector functionality (engulfment by macrophage, neutrophils and dendritic cells), and therefore clearance mechanisms. For instance the murine anti-TcdB recognizing F1 (CAN46G13a, Table 1) was non-neutralizing *in vitro* but fully neutralizing *in vivo*, while the humanized variant (CANmAbB1) was partially neutralizing *in vitro* and only partially neutralizing *in vivo* in the same assays (Table 1 and Fig 2). This suggests that while the kinetics of affinity were similar following the humanization protocol, the effector functionality preserved in the mouse clone was partially lost when grafted onto a human framework, and likely reflects the degree of affinity/function difference between Fc receptors in mice and human IgG Fc [57]. The *in vivo* neutralization ability of CANmAbB1 may be restored again in humans, but this remains to be determined as in current study antibody administrated at equivalent moles, not adjusted for their different clearance rate *in vivo* [58].

Since the neutralization activity of antibodies varies as a function of the genetic and phenotypic heterogeneity of clinical isolates [27,59,60] and is a consideration in further development of *C*. *difficile* anti-toxins, the xCELLigence^®^ assay was developed (Table 2) [61]. In these assays, CANmAbA4 demonstrated significant neutralizing activity in comparison to the positive control, CDA1, across clinical isolates tested, including hypervirulent NAP-1/027 strains (BI-1, BI-6, BI-17). The results suggest this unique epitope is more highly conserved across the tested clinical isolates than the CDA1 epitope, which is also consistent with reduced binding of CDA1 to TcdA from ribotype 027 strains [62]. For the humanized anti-TcdB monoclonal antibodies, neutralization potency across tested clinical isolates was variable. CANmAbB4 and MDX1388 were comparable across the non-NAP1 strains but both were significantly reduced in NAP1 strains. On the contrary, CANmAbB1 proved more consistent to neutralize toxins from both NAP1 and non-NAP1 strains, indicating the divergence within the receptor binding domain and conservation of epitopes within the glucosyltransferase domains between non-NAP1 and NAP1 strains [60]. The highest sequence variability in TcdB from historical strains and hypervirulent (NAP1) strains is mainly found within the C-terminal CROPs and the adjacent region (88% identity between historical and epidemic strains), whereas the N-terminal GTD is more conserved and shows 96% amino acid sequence identity [63]. In the case of CF2 (A-B+ toxinotype, ribotype 017) the marked reduction in the effectiveness of CANmAbB1 neutralization reflects the atypical amino domain of the TcdB (identified epitope of CANmAbB1) which bears high identity between both the VPI 10463 strain, from which it was raised against, and lethal toxin (tcsL) from *C sordellii* [64]. Sequence analysis of the TcdB gene from TcdA−TcdB+ strains identified multiple point mutations in GTD regions, with only 84% homology with that of type strain VPI 10463 [65,66]. Based on these results, an oligoclonal combination of CANmAbA4, CANmAbB4 and CANmAbB1 would be predicted to provide better protection against both hypervirulent and non-hypervirulent clinical strains. Indeed, Davies et al. [35] have demonstrated that co-administration of three mAbs, one anti-TcdA (CA997) and two anti-TcdBs (CA1125 and CA1151) with vancomycin protected hamsters against recurrence in a *C*. *difficile* spore challenge when targeting analogous domains. However, it should also be considered that although MDX1388 does not neutralize TcdB *in vivo* mouse toxicity, it did protect animals against spore challenge in piglet and mouse models [34,54]. Furthermore, it effectively reduced recurrence of NAP1/027/BI hypervirulent strain in humans when combined with CDA1 and vancomycin treatment (8% compared to 32% with vancomycin alone, p = 0.06), especially in patients with more than one previous episode (7% compared to 37% with vancomycin alone, p = 0.006) [16]. Recent clinical studies also demonstrated that anti-TcdB alone was effective to reduce recurrence by about 50% in patients undergoing antibiotic treatment [38,39]. The combinational approach (anti-Tcds and vancomycin), suggests effective management of disease progression is two-fold, whereby vancomycin is reducing *C*. *difficile* infection while providing the anti-toxins the opportunity to neutralize TcdA and TcdB in the blood stream and eventually clear it from the system and re-establish gut microflora.

Testing of CANmAbA4 and CANmAbB4 combinations in an established B1 primary infection model (Fig 3), at two dosages was performed. In these studies high doses (50 mg/kg) of CANmAbA4/CANmAbB4 protected against primary B1 spore infection in 85% of hamsters compared to 50% protection at the low dose (20 mg/kg). The demarcation of B1 infection (severity and increase in frequency) only occurred after 16 DPI for the CANmAb cocktail at the lower dose. This is the typical time frame (~13 DPI) in which others have observed relapse to occur in hamsters treated with vancomycin or anti-toxin antibody combination alone [27,35]. At the end of the experiment, the sera of survived animals were also collected for toxin specific antibody measurement. Although mAbs from 50 mg/kg group were slightly higher those from 20 mg/kg group, the difference was not statistically significant, which is in alignment with the similar survival rate of the two mAb treatment groups. Interestingly, great heterogeneity of the serum anti-TcdA mAb was noticed, which may be derived from the complex kinetics of the humanized mAbs in animals and the variable toxin levels in each individual hamster. A previous study [25] found that circulation titers of i.p. injected human antibodies were lower than expectedn and variable, owing to the transportation with the human antibodies from peritoneum to circulation system [25]. Indeed, some hamsters had non-detectable level of human mAbs after i.p. injection with a high dose of human mAbs. Moreover, the bacterial and toxin levels of each individual hamster may be different; the higher level of toxins could consume more antibodies during the whole study period, which may also contribute to the highly divergent serum anti-TcdA levels. Although the two mAb treated groups didn’t show difference in the survival rate after infection, the 50 mg/kg group did show less body weight loss after infection (P<0.05) compared to the 20 mg/kg group. Perhaps a higher level of mAbs presented in the intestine and bloodstream at the early critical stage of infection [35], which, although didn’t improve the survival rate, did neutralize more toxins and protected the animals against the pathophysical changes. This difference indicates that administration of a higher level of mAbs may be beneficial for the patients by alleviating the symptoms.

Efforts to test CANmAbs in relapse/recurrence models described in the literature [25] were attempted, but we have been unable to replicate these models. Indeed, earlier reports showed that the model is difficult, and recent publications studying anti-toxin antibody functions have only reported on primary infection models [27,35,64]. While a growing body of evidence suggests that passive immunization against *C*. *difficile* TcdA and TcdB in combination with antibiotics is effective, the appearance and evolution of hypervirulent strains (excess Tcd production, increased potency or other mechanisms of actions) and potential for antibiotic resistance in response to widespread use of vancomycin as a primary line of defense have encouraged the development of other therapies [15, 19, 21]. The current study has described the identification and biological characterization of three novel anti-Tcd humanized antibodies with demonstrated neutralization across a broad spectrum of non-NAP1 and NAP1 clinical isolates. Initial combinations of CANmAbA4 and CANmAbB4 were protective against B1 primary spore infection model in a dose dependent manner, and together these attributes make anti-Tcd CANmAbs attractive candidates for development of passive immunotherapy or adjunctive therapies with antibiotics. Fortunately, as the number of anti-Tcd mAbs currently under development increases, the opportunity to create optimal polyclonal combinations against hypervirulent strains may provide the most effective mAb cocktail treatment possible.

## Materials and Methods

### Reagents, *C*. *difficile* strains and cell lines

Polyclonal anti-TcdA and anti-TcdB were obtained from ImmunoPrecise Antibodies (Victoria, Canada) by immunization of rabbits with recombinant *C*. *difficile* Tcds or Tcd fragments, described below. For *in vitro/in vivo* experiments, positive controls CDA1, MDX1388 [25] were constructed and expressed from publically available sequence information (S1 Table). *C*. *difficile* reference strain ATCC43255 (VPI10463) was obtained from American Type Culture Collection (ATCC, Rockville, MD, USA). Nine *C*. *difficile* clinical isolates (Table 2) were kindly provided by Dr. Dale Gerding (Department of Medicine, Hines VA Hospital, Hines, Illinois, USA). As described in the following methods, all *C*. *difficile* strains were cultured on BHIS (brain heart infusion supplemented)/BHIT (brain heart infusion taurocholate) plates for spores and TY broth for toxin production. CT26.wt, HT-29 and Vero cells were obtained from ATCC and cultured/subcultured in optimal media as described by ATCC.

### Recombinant *C*. *difficile* toxins and toxin fragment expression

Recombinant toxin A (rTcdA), toxin B (rTcdB), and subdomains were based on the *C*. *difficile* reference strain, VPI 10463 (S1 Fig). Briefly, *C*. *difficile* toxins A and B (TcdA and TcdB) whole coding sequences were amplified from *C*. *difficile—*strain ATCC43255 (VPI10463) genomic DNA using previous published primers and ligated into pHis1522 shuttle expression vector with a C-terminal poly-His tag (6xHis) to facilitate purification [67]. The expression vectors were then transformed into *Bacillus megaterium* protoplasts (Mo Bi Tec system, Goettingen, Germany) [67,68]. The toxins A and B were expressed in the cells with D-xylose induction and harvested by lysing the cells using a dry ice/ethanol bath. The resulting supernatant was purified on a Ni^2+^ column, eluted by chelation and buffer-exchanged into PBS.

Receptor binding subdomains for TcdA and TcdB (fragment 4; TcdAF4 and TcdBF4) were amplified from *C*. *difficile* ATCC43255 DNA using Easy-A High Fidelity PCR kit-(Agilent Technologies, Mississauga, Canada) and published primers [25]. Amplified fragments were ligated into the pET32a vector in-frame with a poly His-tag to facilitate purification and transformed into *E*. *coli* BL21(DE3) competent cells (Life technologies, Burlington, Canada). Similarly, the glucosyltransferase subdomain (fragment 1; TcdBF1) was also amplified from the same reference strain and recombinantly expressed in pET32a in-frame with a poly His-tag. The BL21 (DE3) cells contain a T7 RNA polymerase and were used to drive expression of the toxin A/B fragment proteins from the T7 promoter in the pET32a plasmid. The toxin fragment 1 and fragment 4 proteins were expressed in the cells with IPTG induction and harvested in the soluble pellet. The soluble pellet was purified on a Ni^2+^ column, eluted by chelation and buffer exchanged into PBS.

Purified toxins and subdomains provided suitable test articles for monoclonal antibody (mAb) characterization. Protein concentrations of purified test articles were determined using Pierce BCA assay (Fisher Scientific, Ottawa, Canada).

### *C*. *difficile* spore culture and concentration calculation

*C*. *difficile* spore culture and purification was based on Sorg’s protocol with slight modification. Briefly, *C*. *difficile* isolates (spores) were cultured anaerobically on BHIS plates [69] containing 0.1% taurocholate (Sigma-Aldrich, Oakville, Canada) (BHI-T plate) for germination and then on BHIS plates for spore formation. Seven days later, the bacterial lawn was re-suspended in ice cold water and subsequently centrifuged. After three additional washes, final pellets were resuspended in 15 ml PBS and heat shocked in a 56°C water bath for 15 minutes. Bacteria preparations were sequentially pelleted and washed three times, in water. Purified spores were diluted in 3 ml PBS and stored as 100μl/vial aliquots in -80°C freezer. Spore concentrations were determined by plating serially diluted stocks on BHI-agar and incubation in an anaerobic chamber for clone counting and calculation.

### Concentration of *C*. *difficile* culture supernatants

To prepare *C*. *difficile* culture supernatants for cytotoxicity assay, *C*. *difficile* isolates were grown on BHI-T plates for 48h anaerobically and a single colony was transferred into TY broth [69] for an additional 4 days culturing in an anaerobic chamber at 35°C. Bacteria cultures were subsequently centrifuged at 4690 x g for 15min. Supernatants were filter sterilized with 0.22 μm low protein binding filters (Millex-GV, Cat# SLGV033RS, Millipore, Etobicoke, Canada) and aliquots were stored at 4°C for subsequent cytotoxicity test and toxin concentration assay by ELISA.

### Humanization of anti-toxin A and anti-toxin B mAbs

With the aid of proprietary software (Discovery Studio from Accelrys/Biovia) both manual and computer assisted methods were used to humanize murine mAbs candidates, described elsewhere [40,41]. Complementarity-determining regions and relevant framework amino acids from the murine heavy and light chains were grafted into the best matching germline allele human IgG1, κ frameworks, synthesized and cloned into pEE6.4 and pEE12.4 vectors (Lonza, USA) as intermediates. Dual gene constructs expressing heavy and light chains were prepared for expression of three fully humanized full length IgG1/κ antibodies. Transiently transfected HEK293F (Invitrogen^™^, Catalog number R790-07, ThermoFisher Scientific, Burlington, Canada) or CHO-S (Gibco^®^, Catalog number A29132, ThermoFisher Scientific, Burlington, Canada) cells or stably transfected CHO-K1SV (Lonza) cells provided the supernatant from which antibodies were purified using Protein A chromatography (GE Healthcare, Missisauga, Canada).

Purified antibodies were identified by SDS-PAGE and western blotting/ELISA against antigens (recombinant toxin or toxin fragments). Further characterization by *in vitro* neutralization, affinity and *in vivo* toxin challenge were also performed where indicated.

### Affinity analysis and Epitope Binning

Biolayer interferometry was used to measure the interactions between whole toxins and the anti-toxin mAbs using the label-free biosensor Octet^®^ QKe (Pall ForteBio Corp, Menlo Park, USA) system. Streptavidin (SA) biosensors (pins) coupled with biotinylated toxins (40 μg/ml) were used to test the interaction in a dilution series from 100 nM to 1.56 nM. The mAbs were reacted with the toxin-coated pins for 10 minutes followed by a dissociation step in PBS for another 10 minutes. The results were then analyzed using ForteBio^®^ Data Analysis.

The epitope binning assay was performed against the previously characterized CDA1 and MDX1388 anti-toxin mAbs [25] to confirm unique epitopes and characterize selected hybridoma/humanized clones. Epitope binning is a competitive binding assay utilized to determine whether a set of mAbs against a target antigen bind similar/overlapping epitopes and share similar functional characteristics [70], therefore mAbs that bind similar epitopes are ascribed into the same epitope families or ‘bins’. In general, biotinylated 1^st^ antibody was captured onto streptavidin biosensors. The bound antibody was then incubated with free recombinant toxin, followed by incubating with free 1^st^ antibody. The antibody-Toxin complex was again incubated with free 2^nd^ antibody. A large nm shift in wavelength will indicate that the test mAb (1^st^ mAb) and the free antibody (2^nd^ mAb) have different epitopes, while minor or no nm shift indicates the two mAbs bind the same/similar epitope.

### *In vitro* neutralization assay

*In vitro* neutralization (IVN) assay for recombinant *C*. *difficile* toxins using CT-26.wt cells (CRL-2638, ATCC, Manassas, VA, USA) was performed to test the neutralization capability of the murine and humanized mAbs against *C*. *difficile* toxins. CT-26.wt cells were grown in RPMI-1640 media (Sigma-Aldrich, Okaville, Canada) (with 10% FBS, 37°C, 5% CO_2_), plated at 3x10^4^ cells/well and allowed to attach to plates (~ 3 hours). The toxin and toxin/mAb mixtures were prepared in microcentrifuge tubes and diluted to the desired concentrations using RPMI-1640 media and left to incubate at room temperature for 1 hour. After removal of media from plates, controls and toxin/mAb mixtures were transferred to designated wells. The plates were incubated an additional 48 hours at 37°C and 5% CO_2_. The WST-1 cell proliferation reagent (Roche Diagnostics, Laval, Canada) was added to each well (10 μl of reagent/100 μl volume in the well) and incubated for 1 hour at 37°C and 5% CO_2_. The plate was shaken for 10 sec and then read at 450 nm. Two blank wells containing only media (no cells) were also included in the plate for background determination. Toxin neutralization is calculated by the formula as below:

% Neutralization = (Sample OD – toxin control OD)/ (Cell control OD – toxin control OD) * 100

### *In vitro* neutralization of culture supernatants from *C*. *difficile* clinical strains

For determining *in vitro* neutralization across a number of clinical isolates, sterile supernatants of cultures were assessed for cytotoxicity *in vitro* (Table 2) to calculate and set up supernatant dilutions which lead to 90% cell death (CT_90_). Based on sensitivity to toxin A or B, HT-29 human colon carcinoma epithelial cells (HTB-38, ATCC, Manassas, VA, USA) and Vero monkey kidney fibroblast cells (CCL-81, ATCC, Manassas, VA, USA) were used for neutralization assays using the xCELLigence^®^ system, respectively. The xCELLigence^®^ is a real-time label-free cell analysis (RTCA) system based on an electronic impedance measurement. When adherent cells are cultured within the custom 96-well plate, cell growth characteristics can be monitored in real-time by changes in electrical impedance within each well. A decrease in impedance may indicate the change of cell size/morphology, cell number, viability and adherence. Specifically, in this study, toxin administration will damage cells and decrease the impedance while neutralization of toxin toxicity with specific antibodies will increase the impedance and align with the cell growth during culturing period. HT-29 cells (8 x 10^3^ cells/well) or Vero cells (7.5 x 10^3^ cells/well) were cultured in corresponding media (ATCC, Manassas, VA, USA), added to a Roche 96-well E–plate^®^ and incubated 4 hours at 37°C. During the incubation, sample mAb serial dilutions were prepared on a 96-well U-bottom plate, and then mixed with the appropriate CT_90_ dilution of *C*. *difficile* culture supernatants. Sample plates were then incubated at 37°C for 60 minutes to allow for toxin/antibody interaction. Cells (HT-29 or Vero) were overlayed with their respective toxin / sample preparations and incubated at 37°C with 5% CO_2_ overlay. Real-time cell impedance was measured at 30 minute intervals over 72 hours using the xCELLigence^®^ system. Data from the final time point was used to generate a 4-parameter logistics curve, and the corresponding EC_50_ (the mAb concentration which accounted for 50% neutralization of the toxicity from the culture supernatants) value was used to determine the neutralizing efficacy of the monoclonal antibody against either TcdA or TcdB. In each single test, the cell culture (without toxin or mAbs) and toxin (bacterial culture supernatants) controls were administrated to ensure the test performed appropriately.

Cells were cultured as outlined in materials and methods. Supernatants derived from independent strains were filtered under sterile conditions and diluted for cytotoxicity (CT_90_) testing to determine EC_50_ for testing neutralizing capacity of selected antibodies. A+/- indicates expression of TcdA, B+/- indicates expression of TcdB, CDT+/- indicates expression of binary toxin. PFGE, Pulsed-field Gel Electrophoresis; REA, Restriction endonuclease analysis.

### Mouse Toxin Challenge Model

The mouse toxin challenge studies were performed at the Richardson Center animal facility of the University of Manitoba under approved protocols (University of Manitoba, Central Animal Care Services (CACS) F10-040). The mouse *in vivo* toxin challenge model was based on previous publications [25] with some modifications. Balb/c mice weighing 20-30g were given 250μg of antibody or vehicle controls intraperitoneal (i.p.) injection 24 hours prior to toxin challenge. Mice received toxin challenge on day 0 with 100 ng of toxin A/mouse or 75ng of toxin B/mouse in 100 μl by i.p. The Tcd selected doses kill 90–100% of animals in 24–48 hours in an unprotected state. Thereafter, mice were monitored over 72–80 hours for signs of abnormality and local or systemic disease. For the first 30 hours after toxin challenge, mice were monitored at 3 hour intervals, followed by 6 hour intervals up to day 3 after toxin challenge. Clinical signs were recorded and animals were ranked as normal, lethargic (ruffled fur, general inactivity, responding to stimulation), abnormal (showed symptoms as hunched posture, isolated, non-responsive to stimulation, not moving, loose skin, deep set/sunken eyes, rapid breathing) and moribund (a combination of three or more of these abnormal symptoms). Moribund animals were euthanized immediately (4% isofluorane anesthesia followed by cervical dislocation) and recorded. Animals were not administered analgesics or anesthetics during the observation period to avoid interference of clinical symptom recording. All observations were recorded and the survival rate was determined for each treatment group.

### Efficacy study of humanized anti-toxin mAbs in hamster primary infection model

The hamster protection study was performed at National Research Council Canada (NRC) under approved protocols (NRC-IBS Animal Care Committee, Approval #2011.21). Groups of female Golden Syrian hamsters (Charles River Laboratories) at the age of 7–8 weeks (weight 100–120 grams) received 4 injections of anti-toxin A and anti-toxin B mAbs with either high (50 mg/kg bodyweight) or low dosages (20 mg/kg bodyweight) each day for four days before infection. On the third day of antibody injection, hamsters were also given 10 mg/kg (bodyweight) of clindamycin to clear gut bacteria flora to enhance *C*. *difficile* spore infection. The last day of antibody injection, hamsters were intragastrically given a dose of 117 B1 spores. The dose was confirmed by plating serial dilutions of inoculums on BHI-T agar plate and incubated in anaerobic chamber for 48 hours. Clinical signs (normal, wet tail, abnormal gait, lethargic) and survival (including euthanization) were recorded twice a day for 22 days along with the body weights measured every two days. To avoid introducing any additional variables into the experiments, animals were not administered analgesics or anesthetics during the observation period. However, animals were monitored more frequently (up to 4 times daily) if any clinical signs of disease were observed. Hamsters remained active, interested in their environment and did not appear to experience any appreciable discomfort until very late in the disease progression. Once physical activity declined and labored breathing was observed, moribund animals (with wet tail, little-to-no locomotor activity and labored breathing) were immediately euthanized (4% isofluorane anesthesia followed by CO_2_ inhalation) and recorded as dead at that time to determine survival rates. At day 22 after infection, all surviving hamsters were euthanized and sera collected and filter-sterilized for anti-toxin antibody level test. Gross necropsy and pathology of surviving animals were also performed and recorded upon termination of the experiment.

Serum was collected prior to antibody injection (Day-3) for all animals and day 22 for all surviving hamsters. Serum specimens were analyzed for the injected toxin-specific antibodies by Bio-Plex^®^MAGPIX^™^ multiplex assay (Bio-Rad, Mississauga, ON, Canada).

### Statistical Analysi*s*

Hamster and mouse survival data was analyzed with log-rank tests. Means of serum antibody were analyzed by one-way ANOVA using GraphPad Prism (vision 5.0, San Diego, California). *In vitro* neutralization data were analyzed by two-way ANOVA modeling using SAS (version 9.3, SAS Institute Inc., Cary, NC, USA).

## Supporting Information

S1 Fig Information of toxins and toxin fragments. Simplified diagram of functional domains demarked by amino acid position, amino acid length, EC50 and deduced molecular weight for toxin A and toxin B from *C difficile* VPI 10463 strain corresponding to recombinant toxins and fragments listed in the table. The recombinant test articles were used for CANmAb characterization, identity, and *in vitro/in vivo* neutralization assays.
(GIF)

S2 Fig Binding activity of humanized mAb variants on *C*. *difficile* toxin A, toxin A fragment 4 and toxin B. The ELISA plate was coated with 400 μg/ml of whole toxin A (TcdA) or whole toxin B (TcdB) and 100 μg/ml of toxin A fragment 4 (TcdA F4). The coats were probed with serially diluted human mAbs and binding was detected with anti-human IgG-HRP antibody. The plate was read at 405 nm after 60 min (**A**) or 15 min (**B**) incubation with substrate. **2A.** The data shown is for 2 μg/ml of mAb on both toxin A coat and toxin A fragment 4 coat. Intermediates shown in this graph include the murine CAN20G2 and humanized CAN20G2 (CANmAbA4). For positive control CDA1 was used and for a negative control M102.4 (an irrelevant mAb) was used. **2B.** The data shows 0.5 μg/ml humanized anti-TcdB mAB (CANmAbB1 and CANmAbB4) activity on *C*. *difficile* toxin B and lack of reactivity against toxin A.
(GIF)

S3 Fig *In vitro* neutralization of toxin activity on CT26.wt cells. **A.** neutralization of toxin A with humanized anti-TcdA mAbs; **B.** neutralization of toxin B with humanized anti-TcdB mAbs.
(TIF)

S1 Table Listing of additional monoclonal and polyclonal antibodies prepared in house for comparisons and positive controls. HC, heavy chain; LC, kappa/light chain. *specificity is reported as whether toxin A (TcdA) or toxin B (TcdB) and the fragment/domain, if known, where F4 corresponds to receptor binding subdomain, and F1 corresponds to glucosyltransferase subdomain as depicted in S1 Fig.
**polyclonal antibodies were raised against rTcdA and rTcdB as described in materials and methods corresponding to full length rTcds depicted in S1 Fig.
(DOCX)

S2 Table Serum levels of humanized antibody levels in hamsters at 22 days after infection (DAI). (DOCX)

## Abbreviations

CDI: *Clostridium difficile* infection
TcdA: toxin A
TcdB: toxin B
mAb: monoclonal antibody
DPI: days post infection
TcdAF4: TcdA fragment 4
TcdBF1: TcdB fragment 1
TcdBF4: TcdB fragment 4
