# Supplementary figures and images for: Novel Clostridium difficile Anti-Toxin (TcdA and TcdB) Humanized Monoclonal Antibodies Demonstrate In Vitro Neutralization across a Broad Spectrum of Clinical Strains and In Vivo Potency in a Hamster Spore Challenge Model
Source: PLoS One. 2016 Jun 23;11(6):e0157970. doi: 10.1371/journal.pone.0157970 (PMC4919053; doi:10.1371/journal.pone.0157970)

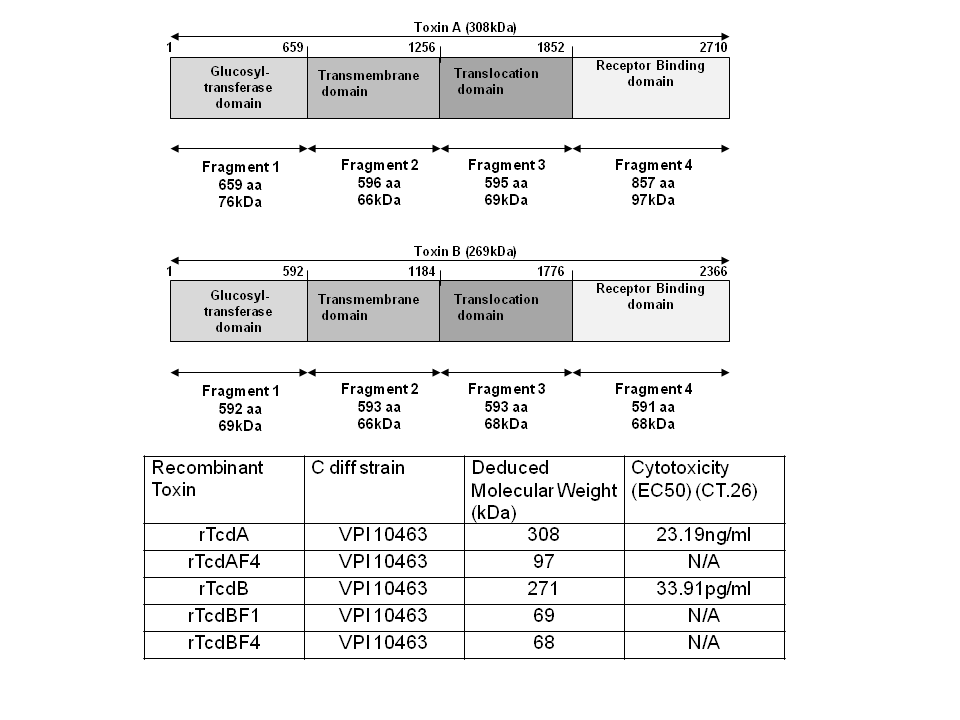

Supplement: S1 Fig — Simplified diagram of functional domains demarked by amino acid position, amino acid length, EC50 and deduced molecular weight for toxin A and toxin B from C difficile VPI 10463 strain corresponding to recombinant toxins and fragments listed in the table. The recombinant test articles were used for CANmAb characterization, identity, and in vitro/in vivo neutralization assays. (GIF) [file pone.0157970.s001.gif]

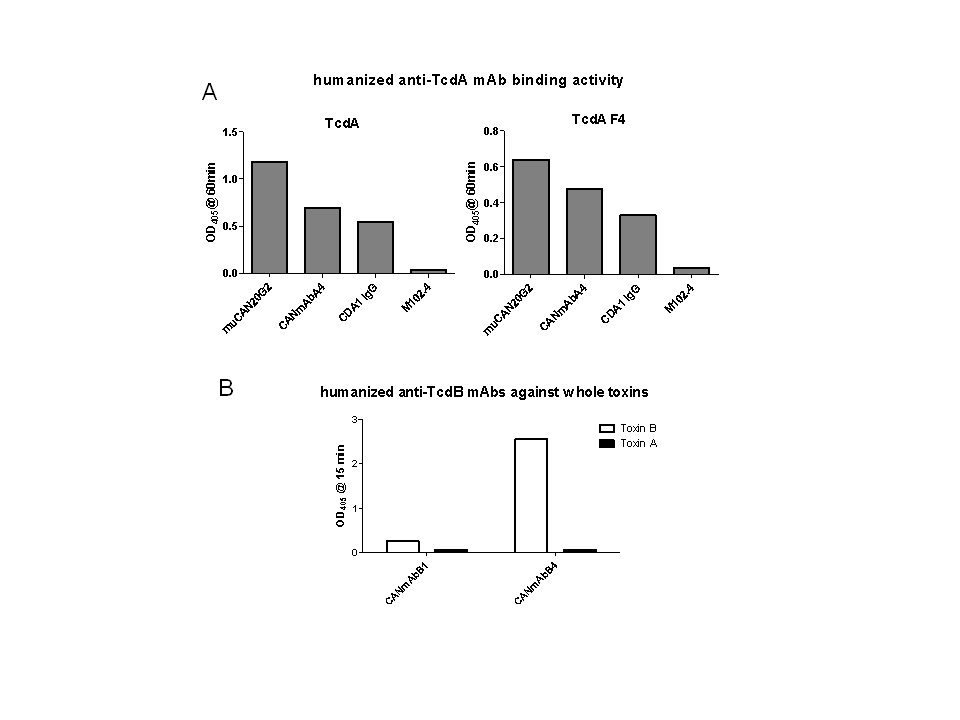

Supplement: S2 Fig — The ELISA plate was coated with 400 μg/ml of whole toxin A (TcdA) or whole toxin B (TcdB) and 100 μg/ml of toxin A fragment 4 (TcdA F4). The coats were probed with serially diluted human mAbs and binding was detected with anti-human IgG-HRP antibody. The plate was read at 405 nm after 60 min (A) or 15 min (B) incubation with substrate. 2A. The data shown is for 2 μg/ml of mAb on both toxin A coat and toxin A fragment 4 coat. Intermediates shown in this graph include the murine CAN20G2 and humanized CAN20G2 (CANmAbA4). For positive control CDA1 was used and for a negative control M102.4 (an irrelevant mAb) was used. 2B. The data shows 0.5 μg/ml humanized anti-TcdB mAB (CANmAbB1 and CANmAbB4) activity on C. difficile toxin B and lack of reactivity against toxin A. (GIF) [file pone.0157970.s002.gif]
