# Supplementary material for: Novel Clostridium difficile Anti-Toxin (TcdA and TcdB) Humanized Monoclonal Antibodies Demonstrate In Vitro Neutralization across a Broad Spectrum of Clinical Strains and In Vivo Potency in a Hamster Spore Challenge Model
Source: PLoS One. 2016 Jun 23;11(6):e0157970. doi: 10.1371/journal.pone.0157970 (PMC4919053; doi:10.1371/journal.pone.0157970)
Supplement: S1 Table — HC, heavy chain; LC, kappa/light chain. *specificity is reported as whether toxin A (TcdA) or toxin B (TcdB) and the fragment/domain, if known, where F4 corresponds to receptor binding subdomain, and F1 corresponds to glucosyltransferase subdomain as depicted in S1 Fig. **polyclonal antibodies were raised against rTcdA and rTcdB as described in materials and methods corresponding to full length rTcds depicted in S1 Fig. (DOCX) [file pone.0157970.s004.docx]

| mAb | Specificity^*^ | Isotype | reference | GenBank Accession#/Published patent |
| --- | --- | --- | --- | --- |
| CDA1 | TcdAF4 | Human IgG1:κ | Babcock et al, 2006 | HC: CS483823, LC:DJ444525 |
| MDX1388 | TcdBF4 | Human IgG1:κ | Babcock et al, 2006 | HC:CS483842,  LC: CS483846 |
| Anti-TcdA pAb^**^ | TcdA | Rabbit polyclonal | ImmunoPrecise Ab Ltd | NA |
| Anti-TcdB pAb^**^ | TcdB | Rabbit polyclonal | ImmunoPrecise Ab Ltd | NA |
