# Supplementary material for: Novel Clostridium difficile Anti-Toxin (TcdA and TcdB) Humanized Monoclonal Antibodies Demonstrate In Vitro Neutralization across a Broad Spectrum of Clinical Strains and In Vivo Potency in a Hamster Spore Challenge Model
Source: PLoS One. 2016 Jun 23;11(6):e0157970. doi: 10.1371/journal.pone.0157970 (PMC4919053; doi:10.1371/journal.pone.0157970)
Supplement: S2 Table — (DOCX) [file pone.0157970.s005.docx]

| **Test Group** | | **Anti-TcdA (nM)** | | | | **Anti-TcdB (nM)** | | | |
| --- | --- | --- | --- | --- | --- | --- | --- | --- | --- |
| mAb dose | Group (Number of values) | mean | +/-SD | min | max | mean | +/- SD | min | max |
| CANmAbA4/CANmAbB4 50mg/kg treatment | Group C (6) | 676 | 695 | 57 | 1992 | 151 | 63 | 68 | 231 |
| CANmAbA4/CANmAbB4 20mg/kg treatment | Group E (4) | 161 | 85 | 88 | 283 | 87 | 26 | 61 | 114 |
